# Identified risk factors and adolescents’ beliefs about triggers for headaches: results from a cross-sectional study

**DOI:** 10.1007/s10194-012-0489-7

**Published:** 2012-10-14

**Authors:** Astrid Milde-Busch, Andreas Straube, Florian Heinen, Rüdiger von Kries

**Affiliations:** 1 Institute of Social Paediatrics and Adolescent Medicine, Ludwig-Maximilians-University, Heiglhofstrasse 63, 81377 Munich, Germany; 2 Department of Neurology, Ludwig-Maximilians-University, Klinikum Grosshadern, Marchioninistrasse 15, 81377 Munich, Germany; 3 Department of Paediatric Neurology and Developmental Medicine, Dr. von Hauner Children’s Hospital, Ludwig-Maximilians-University, Lindwurmstrasse 4, 80337 Munich, Germany

**Keywords:** Headache, Adolescents, Trigger factors, Risk factors, Lifestyle factors

## Abstract

Although there are few studies on adolescents’ beliefs about triggers of headache, none of these compared the associations between perceived and observed triggers. This study aimed at comparing the prevalence of self-perceived and observed risk factors for headache among adolescents. Adolescents from the 10th and 11th grades of high schools answered questionnaires on their headaches and on potential risk factors regarding lifestyle, stress and muscle pain. Individuals reporting to have experienced headache in the preceding 6 months were asked to report what they believed to cause their headache (self-perceived triggers). 1,047 (83 %) of 1,260 adolescents reported headaches. Stress, lack of sleep and too much school work were the most frequently reported self-perceived triggers of headache; in contrast the statistical analysis identified alcohol and coffee consumption, smoking, neck pain, stress and physical inactivity as risk factors for headache. Among individuals with headache, 48 % believed that stress might trigger their headaches, while increased stress scores were only observed in 23 %. In contrast, while 7, 4, 0.3 and 0 % of individuals reporting headache considered consumption of too much alcohol, neck pain, physical inactivity and consumption of coffee might trigger their headache, 56, 51, 36 and 14 %, respectively, were exposed to these risk factors. The prevalence of self-perceived triggers of headache does not correspond to the prevalence of identified risk factors for headaches. While the role of stress was overestimated, the high prevalence of the other confirmed risk factors in adolescents with headache suggests potential for prevention by increasing awareness for these risk factors and appropriate interventions.

## Introduction

Headache is one of the most frequently reported health complaints amongst adolescents [1, 2]. Approximately 5–15 % of the adolescent population suffers from migraine and further 15–25 % from tension-type headache [3–8].

To prevent headache, it is of special importance to gain insight in adolescents’ knowledge about the origin of their headaches. However, only a limited number of studies had investigated the adolescent’s perception of triggers for their headache, especially migraine [9–12]; stress, lack of sleep and environmental factors are amongst the most frequently perceived headache triggers. Even less is known about correlations between self-perception of potential triggers and the actually observed prevalence of risk factors for headache in adolescents: are the affected individuals aware of the risk factors and how many among the adolescents with headache are exposed? The aim of the present study was to investigate self-perceived triggers of headaches among adolescents and to compare these findings with the prevalence of these risk factors among these adolescents reporting headache.

## Methods

The present study was approved by the data safety officer and the Ethics Committee (082-08) of the medical faculty of the Ludwig-Maximilians-University Munich and the Bavarian Ministry for Teaching and Culture. Recruitment and data collection procedures have been described in detail previously [13]. Of all students, who were present at school at the respective day of data collection, 94.8 % agreed to fill in the questionnaire. Questionnaires on headache and associated lifestyle factors of 1,260 students of the 10th and 11th grades (aged between 14 and 20 years) of 11 public grammar schools in Munich, Germany, were available for analysis. Written informed consent was obtained from the parents of the participants. Consent of the participants themselves was assumed when they handed over the completed questionnaire to the study members.

### Headache classification

Subjects who responded positively to the screening question ‘Did you have headache during the last 6 months?’ were all summarized as headache sufferers. They answered further questions regarding characteristics and symptoms of their headaches, duration, frequency and intensity of headache. These questions were constructed according to the criteria of the German translation of the International Classification of Headache Disorders—2nd edition [14]. Based on these items, a classification of migraine, tension-type headache and miscellaneous headache was possible. For the present analyses, no subdivisions between types of headache were made.

### Self-perceived triggers of headache

An open question (‘What do you think, what does trigger your headache?’) allowed adolescents, who reported to experience headache to list any triggers they assumed to be of importance for their headache. A data-driven analysis based on consentaneous assignment by AMB and RvK of participants’ responses to this open question led to a grouping into the following 30 categories of potential triggers for headaches: prevalent diseases (e.g., seasonal infectious diseases, injury), allergy, adverse reaction on medication, genetic predisposition, muscle pain, head injury, other painful movements (e.g., jerkily getting-up), menstrual cramps, problems with the eyes/glasses/contact lenses, problems with braces, prevalent psychological problems (e.g., depression), stress, too much school work, unhealthy diet, few beverages, too much alcohol, smoking or consumption of illicit drugs, too much physical activity, physical inactivity, sexual activity, too many hours with electronic media, too loud music or noise, sensibility against light, too many hours reading, lack of sleep, boredom, lack of fresh air, weather or climatic changes, wet or cold hair, driving in a car.

### Lifestyle and potential risk factors

The questionnaire included a number of items regarding dietary and lifestyle factors, see [15] for a more detailed description. In brief, the following factors were assessed and dichotomized: consumption of alcoholic drinks (‘How much beer, wine and cocktails do you normally drink?’—‘more than one glass of alcoholic drinks per week’ versus ‘one glass or fewer of alcoholic drinks per week’), consumption of coffee (‘How much coffee do you normally drink?’—‘more than one cup’ versus ‘one cup and fewer per day’). Physical activity was determined according the procedure suggested by Kujala et al. [16] on the basis of frequency, duration and intensity of habitual weekly physical activity outside school hours and was dichotomized ‘low’ versus ‘high or moderate’. Furthermore, stress was measured with the trier inventory of chronic stress (TICS) and was dichotomized ‘above-average’ versus ‘normal or below-average’ score in the chronic stress screening scale [13, 17]. Muscle pain was assessed with ‘Do you feel muscle pain in the region of your head, neck or shoulder?’—yes versus no [18]. These risk factors had previously been identified as statistically significantly associated with prevalent headache in this dataset.

### Statistical analysis

Prevalences of all self-perceived triggers of headaches including 95 % confidence intervals were estimated for adolescents who reported headache. Differences in self-perceived triggers between sexes have been analyzed with a χ^2^ test, considering *p* values <0.05 as indicating a statistically significant difference. Prevalences of observed risk factors including 95 % confidence intervals and prevalence ratios between perceived and observed headache risk factors were estimated for adolescents with headache. The evaluations were performed with the SAS software package (version 9.1, SAS Institute Inc. Cary, NC, USA).

## Results

One thousand forty-seven (83 %) of the 1,260 students, who submitted valid questionnaires, reported headache at least once during the past 6 months Migraine was reported by 10 % of the subjects. Tension-type headache was reported by 49 % of the participants. Co-existing migraine plus tension-type headache were found in 20 % subjects. A further 4 % of the subjects reported experiencing miscellaneous headache as demonstrated previously [13].

The proportion of self-perceived triggers for headache in adolescents with headache in the order of magnitude was stress (48 %), few hours of sleep (25 %), too much school work (19 %), too few beverages (14 %), weather or climatic changes (12 %), prevalent diseases (12 %), psychological problems (7 %), too much alcohol (7 %), spent too many hours with electronic media (6 %), too loud music or noise (4 %), no fresh air (4 %), menstrual pain (4 % of girls), muscle pain (4 %), unhealthy diet (3 %), problems with the eyes (2 %), too much physical activity (2 %), other movements (2 %), sensibility against light (1 %), banging the head (1 %), smoking or consumption of illicit drugs (1 %), genetic predisposition (1 %), too many hours reading (1 %), wet or cold hair (1 %), boredom (0.5 %), sexual activity (0.4 %), physical inactivity (0.3 %), allergy (0.2 %), problems with braces (0.2 %), driving in a car (0.1 %), adverse reaction on medication (0.1 %) and consumption of coffee (0 %). Statistically significant differences between the relative reporting frequency between female and male students have been found for the following self-perceived triggers: stress (58 vs. 34 %), too much school work (22 vs. 14 %), weather or climatic changes (15 vs. 9 %), psychological problems (9 vs. 4 %), too much alcohol (2 vs. 13 %), spent too many hours with electronic media (3 vs. 9 %), muscle pain (5 vs. 2 %), problems with the eyes (3 vs. 1 %), too much physical activity (1 vs. 3 %) and sexual activity (0 vs. 1 %).

The prevalence of observed risk factors among the students suffering from headaches (Table 1) decreased in the order of magnitude: drinking more than one glass of alcoholic drinks per week to muscle pain in the head, neck or shoulder, physical inactivity, above-average scores in the chronic stress screening scale of the TICS inventory and consumption of more than one cup of coffee per day. Differences in prevalence of observed risk factors between female and male students in the present study have already published elsewhere in detail [15].

**Table 1:** Proportion of perceived and observed risk factors (with 95 % confidence intervals) and prevalence ratio between perceived and observed risk factors in adolescents with headache

Trigger (*n*)	Prevalence of perceived triggers among adolescents with headache (%)	Prevalence of observed triggers among adolescents with headache (%)	Prevalence ratio between perceived and observed triggers among adolescents with headache
Stress (1,047)	47.6 (44.5–50.6)	22.5 (20.0–25.2)	2.1
Too much alcohol (1,028)	6.6 (5.2–8.3)	55.5 (52.4–58.6)	0.1
Muscle pain (1,047)	3.5 (2.5–4.8)	51.2 (48.1–54.3)	0.1
Physical inactivity (968)	0.3 (0.1–0.8)	35.6 (32.6–38.8)	0.01
Consumption of coffee (1,021)	0	13.6 (11.6–15.9)	0

The prevalence ratios between self-perceived and observed risk factors for headache suggested overestimation of the importance of stress, while the role all other trigger factors was considerably underestimated.

## Discussion

Adolescents with headache assume that stress, lack of sleep, large amount of school work, lack of non-alcoholic beverages, weather and climatic changes would cause their headaches. The prevalence of perceived headache triggers did not correspond to the prevalence of risk factors previously identified in the same cohort among individuals with headache [13, 15, 18]. The mismatch between perceived and observed risk factors for headache in adolescents with headache suggests potential for prevention by increasing awareness for the relevant risk factors and appropriate interventions.

The present findings on self-perceived triggers for headache correspond to some extent to results reported in adolescents with headache from Canada [9] or Thailand [10] and pediatric or adolescent migraine patients from a hospital samples in India [11] and France [12]. In these studies, environmental conditions, stress and lack of sleep were the most commonly perceived triggers for headache. These frequently reported headache triggers are mostly external factors which cannot easily be influenced, while habits that might be amenable to change by the adolescents like health behavior were among the less frequently reported triggers.

The perceived risk factors in this study correspond to the risk factors in a number of studies in adults, e.g., [19–28]. For example, in a population-based study [23], 58 % of adults with migraine and 49 % of adults with tension-type headache reported stress as a trigger for their migraine, 40 and 36 % reported sleep disturbance and 49 and 45 % reported changes in weather conditions as potential triggers, corresponding to 48, 25 and 12 % of the adolescents in the present study reporting stress, lack of sleep and weather conditions.

To our knowledge there is no other study comparing the perception and prevalence of risk factors in adolescents with headache. The issue of a potential mismatch between perceived and observed trigger factors among adolescents with headache could be ideally assessed in the homogenous group of students, since environmental conditions, most importantly school environment, are comparable.

Our data do not allow establishing the triggers for each individual with headache. The statistically significant associations for the observed risk factors may indicate the risk associated with the exposure in absence of bias or confounding. This does not indicate that exposure to this risk factor will always trigger an episode of headache in all exposed individuals reporting occasional headache. A self-perceived trigger may not be the true trigger for headache in the individual either. It is well possible that individuals with headache attribute the true trigger factors for their headaches to other coincident factors or to something believed to be socially more acceptable as a triggering factor. Conversely, in some individuals a firm belief about a perceived risk factor may trigger a headache episode although the trigger could not be identified as a risk factor in observational research (in the sense of a self-fulfilling prophecy). These competing explanations could not be disentangled in the present study nor was this issue addressed in the other studies on trigger factors for headache. Each individual has to identify the most common triggers for his headache episodes. Some guidance, however, can be provided from the analysis of risk factors for headache in populations. If many individuals with headache are not aware of common risk factors which may trigger their headache episodes, raising awareness to these potential triggers may reduce the burden of headache in the population.

### Strength and limitations

The major strength of the present study is the population-based data collection, and the high participation rate, see also [13], although recruitment was confined to a subgroup of high-school students. This may limit external validity since the attendance of high schools in Germany is more prevalent in families with higher than average monthly income.

The assessment of self-perceived causes or triggers of headache is based on an open question, giving the students the opportunity to open freely and independent on researchers’ intentions. As a consequence of this procedure, different responses were grouped into categories by data-driven analysis. Although assignment was based on consensus between two of the authors, it cannot be excluded that different responses of the students could be assigned to other categories. The most frequently reported causes were consistently called by the students as ‘stress’ or ‘lack of sleep’, match with reports from other studies reducing the risk of false categorization.

We indeed do not know which risk factor is relevant for the individual with headache and even less, whether he or she knows about it. For a public health approach, which is the field of epidemiology (where the first and last author have their expertise) the patient is the population: are headache sufferers on average aware of risk factors, which may or may not trigger their individual headache? If on average they are not aware, an intervention to increase awareness might help—not necessarily each individual with his headache, but the average population of adolescents with headache.

It has to be taken into consideration that our study, similarly as most of the previously published studies on risk factors for headache in adolescents, could only establish associations, but no causal relationships.

## Conclusions

The prevalence of self-perceived triggers of headache does not correspond to the prevalence of identified risk factors in adolescents with headaches. While the role of stress was overestimated, the high prevalence of the confirmed risk factors neck pain, physical inactivity, consumption of alcohol and coffee in individuals with headache suggests potential for prevention by increasing awareness for these risk factors and appropriate interventions.
